# Child Art

**Published:** 1957-10

**Authors:** D. E. Milner

**Affiliations:** Principal, West of England College of Art


### CHILD ART

BY

D. E. MILNER., O.B.E., A.R.C.A., R.W.A.
*Principal, West of England College of Art*

Children, to be healthy, must be both mentally and physically well, and good health includes the preservation of sight and the development of understanding through visual stimulus. Art is the offspring of visual stimulus, and arts and handicrafts can contribute to the well-being of both children and adults, particularly in a mechanically ordered world which tends increasingly to deprive human beings of their natural urge to create and make things for themselves.

The belief that art can make a positive contribution to the mental development of the child has been growing since the end of the first world war, and in 1953 an exhibition at a UNESCO conference of twenty-two nations on the subject of Child Art, held at Bristol University, showed clearly that much thought and experiment had been given to the educational value and practice of art by children.

Art, which is an expression of creative talents is not capable of being tested effectively by the yardstick of examination, and for that reason it often met with neglect in the past, particularly in grammar schools.

It is satisfactory, however, to be able to look round many schools today and to see that Art is now regarded as one of the subjects by which character and personality can be moulded and developed through its practice, and also, that through a study of its history, a clearer view of world history may be gained.

### THE ART OF A CHILD

There is much difference between the art of the professional artist, who logically develops and carries out his work to its pre-determined conclusion and the art of the child whose work is the expression of a spontaneous desire to draw, paint, create or make, usually quite unhampered by the difficulties of technique or theory. The desire to master technique comes soon enough, but not before the age of ten in most children, and it is important that the teacher should realize this; for the effect of a teacher who tries to impose professional or adult techniques of drawing and painting upon children who are not ready for them can be catastrophic. The great danger in all teaching lies in the assumption that the only reason for the pupils' presence in class is that they should be intelligently receptive: that they should be conditioned, indeed, into becoming receptacles into which useful knowledge can be poured and stored. Fortunately, it is now being realized that education should also include the experience and development of perception, and that what has been intelligently perceived may promote and contribute to aesthetic and creative exercise.

As a child's awareness of the sensations of sight and hearing become more pronounced, so, if he is properly guided, will his powers of visual expression develop. Most of us who are parents are rightly concerned that our children have eyes that are sound physically, but I doubt whether many fathers or mothers are unduly concerned about the problem of teaching their children how to *use* their eyes intelligently and still fewer who have even considered that their children's eyes can be used creatively.

### SENSE OF ACHIEVEMENT

The value of art as an exercise for a child lies in the fact that it is the application of knowledge gained through the eyes; but it is enhanced also by the fact that it involves the stimulating use of materials, colours, brushes, paper, and so on. The child is thus given an immediate sense of achieving something, and because his achievement is concerned with his own private and personal visual impressions, he is developing his own personality. Unconsciously, perhaps, he is also submitting to a discipline which has its own beneficial effects, and as he grows older, if he does not lose his visual stimulus, that discipline will become increasingly valuable, for it is the discipline of craftmanship.

The surgeon and the dentist know as well as the artist the real value of craftmanship not only in respect of the job done but to the doer himself.

### PLACE OF THE TEACHER

I would stress the importance of the teacher, for teaching itself is a creative art. We all know that children are susceptible to outside influences, and that the weight of a teacher's authority can easily be greater than that of the parent—so much the more carefully, therefore, should his influence be applied.

One of the first things a teacher must do is to encourage the desire to create and to use materials properly and with conviction. The idea that is sometimes held, that all that is required is to supply children with paper and paint and then wait for the miracle to happen is entirely erroneous; but this is not to suggest that the diametrically opposite approach of painting by rule and order is correct.

It is the task of the teacher to ensure that each child works consciously as an artist and it is not always easy to create the right conditions under which this may be achieved.

The teacher must believe with conviction that which the child artist believes instinctively, namely, that reality is fundamental to all true art. By this I mean a personal vision expressed with belief and enthusiasm. Realistic vision may approximate to the true appearance of what is being painted, but it may equally be something more symbolic or even near to the completely abstract. It is necessary in these days to remember that the photograph is not the yardstick of visual reality, nor is a photograph a work of art just because it is an image. The reality of art lies in the reality of its creation or its design to the artist who makes it. It is the reality of the child's own work to himself that is so important, and not the degree to which that reality approximates to the adult's vision. Indeed, it is the child's conception of reality, which so often ignores true perspective, that appears so charming to the adult spectator.

### ABSTRACT AND IMAGE

Nature is the material of the artist, and it is the impact of nature upon the artist's senses that promotes the making of a work of art. At times the result of that impact may take the form of a faithful representation of appearances, but it may equally take the form of an almost abstract arrangement of colour such as might be seen in a sunset observed from a cliff top, with no intermediary objects in view, or it may take the form of the symbolism that has been used in folk art from the beginnings of civilization. Symbolic art and abstract art are not necessarily any less or any more artistically valuable than realistic art, and in the special realm of child art the same applies.

The child, the unselfconscious artist, is to some extent at a disadvantage compared with the trained artist, but provided he has the spirit to respond to his visual impulses he may possess certain advantages. For instance, he does not worry over much about perspective nor with size relationship. He sometimes disregards colour relationships provided that what he is doing is satisfactory to his creative urge, but we may find of course that colour blindness is the cause, a defect that is more common than many lay people imagine.

Owing to the domination of the photograph over our day to day visual responses, we are apt to forget that before the camera was invented it would have been considered quite correct to give prominence to an important figure in a painting or a piece of sculpture by making it larger than its surrounding figures or by exaggerating it in one way or another. A child, whose mind has not yet been conditioned by the photographic image, will often respond to such reasoning. I do not want to imply that child art should be kept childish beyond its normal span. As children grow older they usually grow more objective in their approach to drawing and painting, and in adolescence the problems of teaching become in consequence very much greater.

### THE JOY OF IT

In the practice of art by children, and even by adults, the pleasure of using material, of the feel of paint, or the rhythmical use of the hands is contributory to success. There is an enormous satisfaction to be gained out of feeling that colour is being applied as the mind directs, or that the hand is drawing in harmony with the object drawn. In pattern making, for instance, there are many exercises that demonstrate the value of rhythm in the physical aspects of painting to a degree that may not be generally appreciated.

While the response to art of the average child between the ages of five to twelve years is one of happy confidence, the adolescent girl or boy often faces the problem of drawing and painting or even of craft work with difficulty or even distaste.

### AND FEARS

Fear is the chief source of most of the discouragement felt by adolescent boys and girls. They naturally begin to compare their work with that of adults, and their immature critical faculties cause them to look for qualities in art that are either false or beyond their grasp. They often attempt a more sophisticated expression based on glossy magazine cover design, or they ape the photograph with equally disastrous results.

The conflict which may appear during adolescent years between artistic and creative instincts on the one hand and the acquirement of knowledge on the other can be resolved by the sympathetic teacher. Teachers have the task of mitigating the fears of adolescence, and as the good teacher knows that the success of a work of art, depends upon the intensity of the visual and creative experience, and *not* upon a special kind of dexterity, his main task is to give confidence to his growing pupils.

Once confidence has been gained, much of the difficulty of ensuring the continuity of artistic expression right into adult years will have been solved.

### ADOLESCENCE

The needs of individual adolescents vary very much more than those of younger children, for while confidence and personality develop steadily in some boys and girls as individuals, others need companionship. Some develop best by working individually and alone, while others can only make progress by working in a group.

A great deal of group art work is now being undertaken in schools with amazingly good results, and in Bristol there are examples of large decorative projects successfully carried out by groups of children working together, often under the leadership of one child who the rest may feel has the necessary creative determination.

### EDUCATIONAL VALUE OF ART TODAY

The contribution of art to the development of civilization, its place in history showing us how nations have lived, its place in present day life as a means of awakening discrimination and taste—all these are so important in the education of children; yet, on the whole, how little are they regarded compared with the development of skills for purely material ends!

We are living in a world of mass production, of television, gramophones, picture reproductions and machine-made art. I am not for a moment going to condemn these wholly as the works of the devil, but there is no doubt that day by day they are gradually making it less and less necessary for ordinary men and women or boys and girls to give expression to the creative instincts with which they were born.

Not every boy or girl can expect to become an artist, but art in education can be an essential aspect of the development of well being in children, Not only children but men and women can find that purchased pleasure becomes unsatisfying. They may find also that a sense of achievement and a release from stress that lies in the practice of arts or crafts are benefits that are not to be measured in pounds, shillings and pence.

